# Dual integrin αvβ 3 and NRP-1-Targeting Paramagnetic Liposome for Tumor Early Detection in Magnetic Resonance Imaging

**DOI:** 10.1186/s11671-018-2797-6

**Published:** 2018-11-27

**Authors:** Yin Song, Wei Li, Shuyan Meng, Wei Zhou, Bo Su, Liang Tang, Yinmin Zhao, Xiaoyan Wu, Dazhi Yin, Mingxia Fan, Caicun Zhou

**Affiliations:** 1 Department of Medical Oncology, Shanghai Pulmonary Hospital and Thoracic Cancer Institute, Tongji University School of Medicine, No. 507, Zheng Min Road, Shanghai, 200433 People’s Republic of China; 2 Central Laboratory, Shanghai Pulmonary Hospital, Tongji University, Shanghai, 200433 China; 3 Department of Radiology, Shuguang Hospital Affiliated to Shanghai University of Traditional Chinese Medicine, Shanghai, 201203 China; 4 Shanghai Key Laboratory of Magnetic Resonance and Department of Physics, East China Normal University, Shanghai, 200062 China

**Keywords:** Dual-targeted, MRI, αVβ3-integrin, Neuropilin-1, Tumor imaging

## Abstract

Enhanced MRI (magnetic resonance imaging) plays a vital role in the early detection of tumor but with low specificity. Molecular imaging of angiogenesis could efficiently deliver contrast agents to the tumor site by specific targeted carriers. We designed and synthesized dual-targeted paramagnetic liposomes functionalized with two angiogenesis-targeting ligands, the αVβ3 integrin-specific RGD (Arg-Gly-Asp) and the neuropilin-1 (NRP-1) receptor-specific ATWLPPR (Ala-Thr-Trp-Leu-Pro-Pro-Arg) (A7R). These liposomes were proved to be in the nanoparticle range and demonstrated to effectively encapsulate paramagnetic MRI contrast agents Gd-DTPA (gadolinium-diethylenetriamine pentaacetic acid). T1 relaxivity of various liposome formulations was lower than pure Gd-DTPA but with no statistically significant difference. In vitro cellular uptake and competitive inhibition assay showed the higher binding affinity of dual-targeted liposomes to HUVECs (human umbilical vein endothelial cells) and A549 cells compared with pure Gd-DTPA, non-targeted, and single-targeted liposomes, which was proved to be mediated by the binding of RGD/ανβ3-integrin and A7R/NRP1. For MR imaging of mice bearing A549 cells in vivo, dual-targeted liposomes reached the highest SER (signal enhancement rate) value with a significant difference at all experimental time points. It was about threefold increase compared to pure Gd-DTPA and non-targeted liposomes and was 1.5-fold of single-targeted liposomes at 2 h post injection. The SER was lowered gradually and decreased only by 40% of the peak value in 6 h. Dual-targeted liposomes were likely to exert a synergistic effect and the specificity of delivering Gd-DTPA to the tumor site. Therefore, dual-ανβ3-integrin-NRP1-targeting paramagnetic liposome with a RGD-ATWLPPR heterodimeric peptide might be a potent system for molecular imaging of tumor.

## Introduction

Magnetic resonance imaging (MRI) plays a vital role in detecting solid tumors at an early stage because it provides a better spatial resolution than computed tomography (CT) and positron emission tomography (PET) [1]. Moreover, the application of paramagnetic contrast agents such as gadolinium-diethylenetriamine pentaacetic acid (Gd-DTPA) further improves the signal-to-noise (S/N) ratio [2, 3]. However, low specificity of MRI in the early diagnosis of tumors is still an issue.

Liposome can carry hydrophilic “cargo” in the aqueous environment with integrated amphiphilic or hydrophilic agents in its lipid bilayer. Liposome protects its contents from interacting with components in the plasma, achieving a prolonged biological half-life of hydrophilic “cargo”; hence, liposome is used more frequently as a carrier of contrast agents in MRI [4–6]. Furthermore, by conjugating peptides, antibodies, aptamers, or small molecules to lipid bilayer [7–9], the properties of liposome surface could be modified to enhance their activity in “cargo” delivery or targeting to specific cells and tissues [10, 11]. For targeting tumor, peptides are commonly used to attach to proteins such as ανβ3-integrin, vascular endothelial growth factor receptor (VEGF-R), and galectin-1 which are overexpressed in both endothelial cells and a myriad of tumor cells [12–14]. By targeting and interfering these proteins, the process of angiogenesis in solid tumors was expected to be blocked, subsequently to inhibit the tumor cell growth and metastasis [15–18]. These overexpressed proteins are also attractive candidates for molecular imaging to identify tumor localization at its early stage [19–21].

Nevertheless, the heterogeneous expression of various receptors for tumor angiogenesis could interfere with the targeting ability of single-targeted probes [22]. To solve the problem, simultaneous targeting of dual receptors may expand the population of recognized cells and provide strengthened binding affinity via conjugations of two different ligands to the receptors on the same cell surface. Theoretically, dual-targeted carriers could efficiently deliver more contrast agents to the tumor site for molecular imaging [23–26].

In our previous study, paramagnetic liposomes with conjugated Arg-Gly-Asp (RGD)-lipopeptide could effectively deliver a sufficient amount of contrast agents into tumor [27]. Thus, we hypothesized that by targeting two molecules simultaneously in tumor angiogenesis, e.g., ανβ3-integrin and neuropilin-1, could enhance the signal of paramagnetic liposome-based MR imaging of tumor. Two high-affinity ligands of RGD for ανβ3-integrin and Ala-Thr-Trp-Leu-Pro-Pro-Arg (ATWLPPR, A7R) for neuropilin-1 (NRP1, a VEGF-R co-receptor) were functionalized to the liposome by conjugating with 6-aminohexanoic acid (C6)-palmitic acid (Pal). These dual-targeted Gd-DTPA-encapsulated liposomes were evaluated by comparison with pure Gd-DTPA, non-targeted, and single-targeted liposomes by using in vitro and in vivo assays.

## Materials and Methods

### Chemicals

Egg phosphatidylcholine (C40H82NO9P, egg PC, MW 775 Da) and N-(carbonyl-methoxypolyethylene glycol-2000)-1,2-distearoyl-sn-glycero-3-phosphoethanolamine (mPEG2000-DSPE, MW 2788 Da) were obtained from Avanti Polar Lipids (Alabaster, AL, USA), and cholesterol (C27H46O, MW 386 Da) was obtained from Bio Basic (Ontario, Canada). Gadopentetic acid dimeglumine salt injection (Gd-DPTA, Magnevist) was purchased from Bayer Schering Pharma (Berlin, German). The peptides and conjugates were synthesized by Yishengyuan (Shanghai, China).

### Peptides and Conjugates

Three peptides include dual-targeted peptide P1 (GARYCRGDCFDATWLPPR, MW 2435 Da), single-targeted peptide P2 (GARYCRGDCFDG, MW 1670 Da), and single-targeted peptide P3 (ATWLPPR, MW 1191 Da). Peptides were conjugated with 6-aminohexanoic acid (C6)-palmitic acid (Pal), and the targeting peptides of Pal-C6-P1, Pal-C6-P2, and Pal-C6-P3 were all synthesized using fluorenylmethoxy carbonyl (FMOC) solid-phase synthesis chemistry. The purity of peptide was confirmed to be > 90% by HPLC.

### Liposomes Preparation

Liposomes were prepared by using the thin film hydration method. The composition of liposomes was egg PC/cholesterol/mPEG2000-DSPE at a molar ratio of 1.85/1/0.15. Three components were mixed and dissolved in chloroform, the solvent was evaporated at 37 °C, and a thin film formed at the bottom of round flask. The thin film was dried overnight at room temperature. For the preparation of targeted liposomes, peptides were dissolved in dimethyl sulphoxide (DMSO) and then diluted in chloroform (final DMSO concentration to be 1%). The liposome of P1-Gd-LP, P2-Gd-LP, P3-Gd-LP, and P2/P3-Gd-LP added Pal-C6-P1 to a 4.5 μg/μmol peptide/total lipid ratio, Pal-C6-P2 to a 3 μg/μmol peptide/total lipid ratio, Pal-C6-P3 to a 2.5 μg/μmol peptide/total lipid ratio, Pal-C6-P2, and Pal-C6-P3 to 3 and 2.5 μg/μmol peptide/total lipid ratios, respectively. In preparation of paramagnetic liposome, the thin film was hydrated with Gd-DTPA aqueous solution, then the suspension was extruded ten times sequentially through 0.4 μm, 0.2 μm, 0.1 μm polycarbonate membranes by mini extruder (Avanti Polar Lipids, USA). Unencapsulated Gd-DTPA were removed by centrifugation at 10,000×*g* at − 4 °C (Avanti J-E, Beckman Coulter, CA, USA) through ultrafiltration centrifuge tubes of 100,000 MWCO, Amicon Ultra-15 (Millipore, MA, USA). The final suspension including non-targeted liposomes (Gd-LP), dual-targeted liposomes (P1-Gd-LP), single-targeted liposomes (P2-Gd-LP or P3-Gd-LP), and mixed-single-targeted liposomes (P2/P3-Gd-LP) were stored at 4 °C under nitrogen.

### Liposome Characterization

The size distribution of the prepared liposomes was determined by using submicron particle size analyzer (Zetaplus, Brookhaven Instruments, USA). The morphology of liposomes was observed by transmission electron microscope (TEM, JEM-1230, JEOL, Tokyo, Japan) in staining of uranyl acetate. The concentration of gadolinium was determined by inductively coupled plasma optical emission spectrometer (ICP-OES, Optima 7000DV, PerkinElmer, USA).

### Measurement of T1 Relaxivity

The T1-weighted images of the liposomal suspension were obtained using a 3.0 Tesla nuclear magnetic resonance analyzer (Philips, GE, USA). Pure Gd-DTPA, Gd-LP, P1-Gd-LP, P2-Gd-LP, and P3-Gd-LP solution were diluted respectively with phosphate buffered saline (PBS) to a gadolinium concentration of 1 × 10^−3^ mM to 1 × 10 mM Gd/L. For measuring the longitudinal relaxation T1 (s), an inversion recovery spin-echo (STIR) sequence was used with ten different inversion times (TI) ranging from 200~9000 ms, and other scanning parameters were as follows: repetition time (TR) 10000 ms, echo time (TE) 7.6 ms, the field of view (FOV) 2 × 2 cm^2^, matrix size 320 × 320 and a slice thickness of 5.0 mm. The T1relaxivity (s^−1^ mM^−1^) could be obtained through the following formula: r1 = (R1obs-R1m)/C. R1obs and R1m were the relaxation rates R1 (s^−1^) of the preparations and the corresponding matrix, and C was the concentration of gadolinium (mM).

### Cell Lines and Culture

A549 cells (human adenocarcinoma cell) and HUVECs (human umbilical vein endothelial cells), both expressing ανβ3-integrin receptor family and neuropilin-1 receptors, were provided by the Cancer Institute of the Tongji University School of Medicine (Shanghai, China). Cells were cultured in Dulbecco’s Modified Eagle Media (DMEM, Invitrogen, USA) supplemented with 10% neonatal bovine serum and 100 U mL^−1^ penicillin and 100 μg mL^−1^ streptomycin at 37 °C, 5% CO2. Cells were cultured in 6-well plate until 80–90% confluence in assays.

### Cellular Uptake and Competitive Binding

Five paramagnetic liposomes including Gd-LP, P1-Gd-LP, P2-Gd-LP, P3-Gd-LP, and P2/P3-Gd-LP with gadolinium concentration of 10 mM were administered to HUVECs and A549 cells at 37 °C for 4 h. Following two times of PBS rinse, the nitric acid was added and then, the cells in media were nitrated at 65 °C overnight. In the competitive binding assay, the corresponding free peptides were simultaneously incubated with conjugated liposomes and cells. The final gadolinium concentrations were determined by ICP-OES.

### MRI Capability of Detection In Vivo

All animal procedures conform to the Guide for The Care and Use of Laboratory Animals. The 4-week-old female BalB/C nude mice (SLAC, Shanghai, China) were injected subcutaneously with A549 cells (1 × 10^−4^ cells per mouse) at the right flank. When the size of the tumor reached 50–100 mm^3^, the tumor-bearing mice were randomly assigned into five groups (each *n* = 5). For MR imaging, the mice were anesthetized with a peritoneal injection of 10% urethane (m/v) and scanned at a 1.5 Tesla nuclear magnetic resonance analyzer (Philips, GE, USA). First, T2-weighted images were acquired to localize the tumor using the procedure as follows: TR = 7.3 ms, TE = 2.7 ms, FOV = 12.0 × 12.0 cm^2^, slice thickness = 2 mm, matrix size = 256 × 128. Before intravenous injection of contrast agents, T1-weighted images were acquired for plain scanning by the spin-echo sequence: TR = 420 ms, TE = 14.8 ms, FOV = 12.0 × 12.0 cm^2^, slice thickness = 2.0 mm, matrix = 256 × 128, then six consecutive slices were observed. Following injection of paramagnetic contrast agents, T1-weighed images were acquired at time points of 0.5, 1, 2, 4, and 6 h. The regions of interest (ROIs) of the tumor and hind limb muscle areas in MR images were delimited, and the mean signal intensity (SI) in ROIs before and after contrast injection was used to estimate the SER as described in our previous study [27].

### Statistical Analysis

Data were expressed as mean ± SD, and the multiple comparisons among means were analyzed with one-way ANOVA by SPSS 22.0 software. Two-tailed *P* value less than 0.05 was considered as significant.

## Results

### Liposome Characterization

All the agents loaded liposomes with non-, single-, and dual-targeted peptides were shown in round or oval shape of similar size surrounding clear lipoid structure under TEM. These nanoparticles were less than 100 nm in diameter, and the zeta potential ranged from − 15 mv to − 60 mv measured by zeta potentiometer. The mean sizes of Gd-LP, P1-Gd-LP, P2-Gd-LP, and P3-Gd-LP were 87.75 ± 0.87 nm, 103.50 ± 1.21 nm, 89.91 ± 1.46 nm, and 89.90 ± 1.18 nm, respectively.

### T1 Relaxivity of Dual-Targeted Liposome

**Fig. 1:**
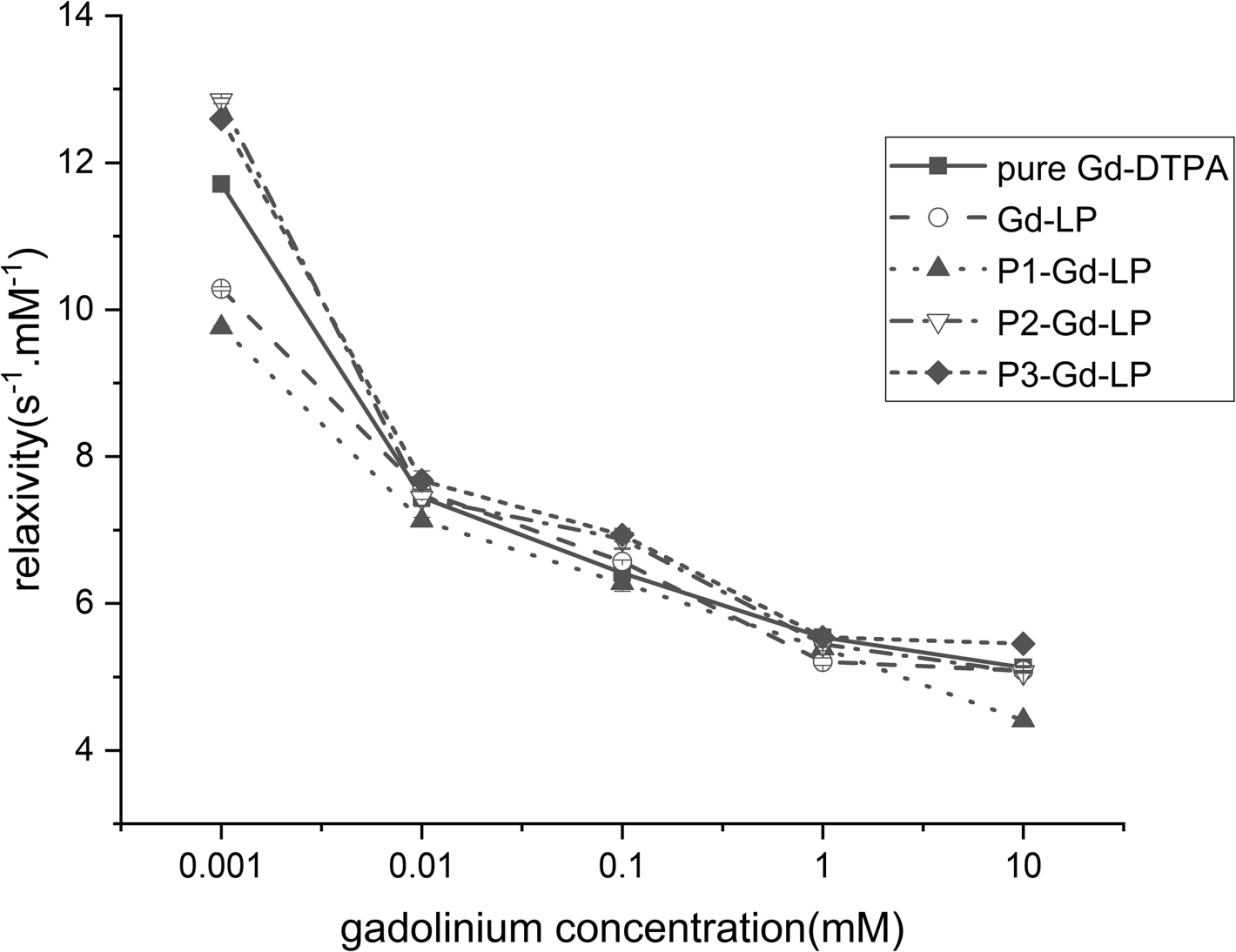
T1 relaxivity (s^−1^ mM^−1^) of pure Gd-DTPA, Gd-LP, P1-Gd-LP, P2-Gd-LP, and P3-Gd-LP solution measured in different gadolinium concentrations (mM). The data represent the mean ± standard deviation (*n* = 3), (*P* > 0.05)

Pure Gd-DTPA possessed the highest relaxivity value in five groups but was not different from the other four types of liposomes (*P* > 0.05) (Fig. 1), indicating that the addition of lipid and peptide compositions made little effects on relaxivity of encapsulated Gd-DTPA. Thus, it suggested that non-, single-, and dual-targeted liposomes encapsulated with Gd-DTPA could be expected to have sufficient capability for molecular imaging.

### Cellular Uptake and Competitive Binding

**Fig. 2:**
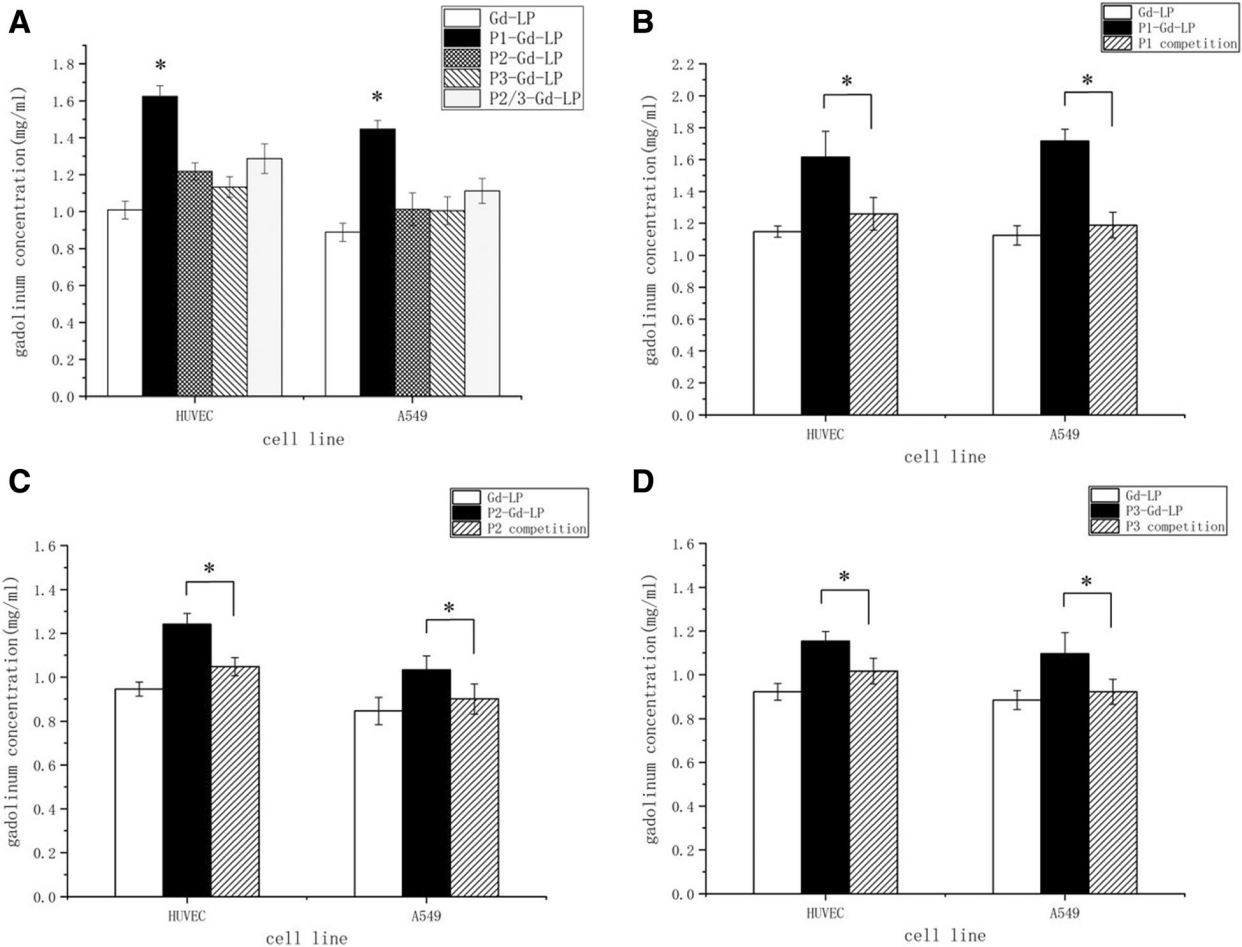
**a** Cellular uptake experiments of Gd-LP, P1-Gd-LP, P2-Gd-LP, P3-Gd-LP, and P2/P3-Gd-LP in A549 cells and HUVECs. **b**-**d** Cellular competition study of P1-Gd-LP, P2-Gd-LP, and P3-Gd-LP, with P1, P2, and P3 respectively added to inhibit receptors in the competition groups. *P < 0.05, vs the other groups

The gadolinium concentration in the dual-targeted liposomes group was higher than other formulas in the cellular uptake study. Compared with non-targeted liposomes, the gadolinium concentration of dual-targeted group raised by 50% (Fig. 2a). It was up to 20% increase in gadolinium concentration of single-targeted liposomes groups. Moreover, the gadolinium concentration of mixed single-targeted liposomes (P2/P3-Gd-LP) was significantly lower than the dual-targeted liposome.

The gadolinium concentrations in targeted liposomes groups were significantly decreased in competitive binding with ligands P1, P2, or P3 to ανβ3-integrin and/or the neuropilin-1 receptors. The cellular uptake in competitive groups was close to that of the non-targeted liposome (Fig. 2b–d and Table 1). These data indicated that the dual-targeted liposome had the best tumor-targeting ability among these groups which was mediated by the binding of RGD/ανβ3-integrin and A7R/NRP1.

**Table 1:** Cellular competition study of P1-Gd-LP, P2-Gd-LP, and P3-Gd-LP, with P1, P2, and P3 respectively added to inhibit receptors in the competition groups. The data of all experiments were expressed by the mean ± standard deviation (*n* = 5)

Gd concentration (mmol/l)	HUVEC				A549			
	Gd-LP		P1-Com		Gd-LP		P1-Com	
P1-Gd-LP	1.15 ± 0.04	*P* < 0.001	1.26 ± 0.10	*P* = 0.001	1.12 ± 0.06	*P* < 0.001	1.19 ± 0.08	*P* < 0.001
	1.62 ± 0.16		1.62 ± 0.16		1.72 ± 0.07		1.72 ± 0.07	
Gd-LP	–		1.26 ± 0.10	*P* = 0.227	–		1.19 ± 0.08	*P* = 0.029
			1.15 ± 0.04				1.12 ± 0.0	
	Gd-LP		P2-Com		Gd-LP		P2-Com	
P2-Gd-LP	0.95 ± 0.03	*P* < 0.001	1.05 ± 0.04	*P* < 0.001	0.85 ± 0.06	*P* = 0.001	0.90 ± 0.07	*P* = 0.007
	1.24 ± 0.05		1.24 ± 0.05		1.03 ± 0.06		1.03 ± 0.06	
Gd-LP	–		1.05 ± 0.04	*P* = 0.045	–		0.90 ± 0.07	*P* = 0.202
			0.95 ± 0.03				0.85 ± 0.06	
	Gd-LP		P3-Com		Gd-LP		P3-Com	
P3-Gd-LP	0.92 ± 0.04	*P* < 0.001	1.02 ± 0.06	*P* < 0.001	0.88 ± 0.04	*P* < 0.001	0.92 ± 0.06	*P* = 0.002
	1.15 ± 0.4		1.15 ± 0.4		1.10 ± 0.10		1.10 ± 0.10	
Gd-LP	–		1.02 ± 0.06	*P* = 0.075	–		0.92 ± 0.06	*P* = 0.401
			0.92 ± 0.04				0.88 ± 0.04	

### MR Image Analysis

**Fig. 3:**
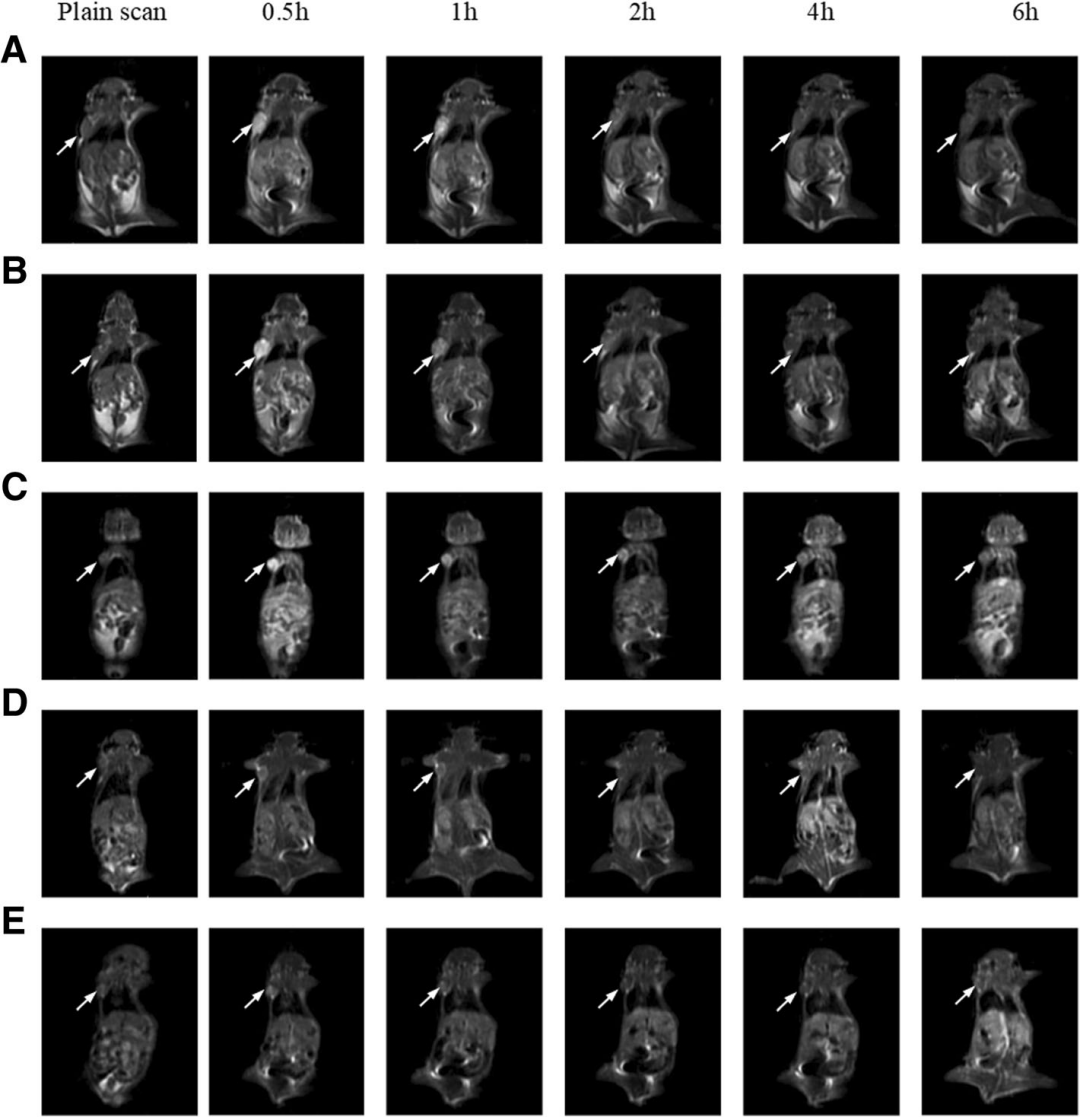
MR images of tumor-bearing mice before and after injection with different contrast agents in different time points. **a** pure Gd-DTPA. **b** Gd-LP. **c** P1-Gd-LP. **d** P2-Gd-LP. **e** P3-Gd-LP

**Fig. 4:**
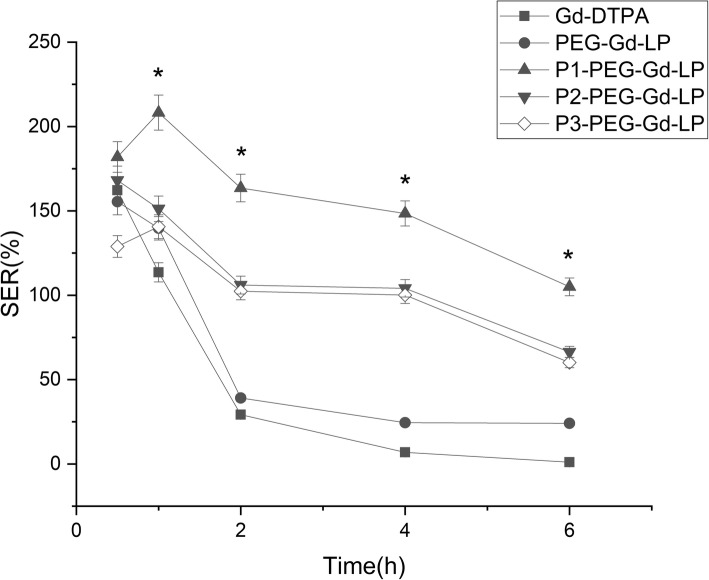
Determination of SER in different time points with an injection of pure Gd-DTPA, Gd-LP, P1-Gd-LP, P2-Gd-LP, and P3-Gd-LP. *N* = 3, and *P < 0.05 P1-Gd-LP vs the other four groups

Conventional liposomes and liposomes with encapsulated gadolinium contrast agents were injected into tumor-bearing mice to evaluate the effect on signal enhancement of tumor in MRI (Fig. 3). In terms of SER, the imaging effects of pure Gd-DTPA and non-targeted liposome groups were similar (Fig. 4). The SER peaked in 1-h post injection and dropped sharply in the following 6 h, whereas the single- and dual-targeted liposome indicated different enhancement patterns with the two groups above. The SER peaked at 1 h but it descended slowly from 2 to 6-hour time point. Among them, dual-targeted liposomes reached the highest SER value with statistic significant at all time points. It was about threefold increase compared to pure Gd-DTPA and non-targeted liposomes and was 1.5-fold of single-targeted liposomes at 2 h post -injection. The SER was lowered gradually and decreased only by 40% of the peak value in 6 h.

## Discussion

Small liposome particles especially those with a diameter less than 100 nm tend to extend the biological half-life with the enhanced permeability into the solid tumor and consequently accumulated in local tumor tissue [4]. We successfully constructed non-, single-, and dual-peptides modified liposomes with the diameters in the nanoparticle range and demonstrated these liposomes could effectively encapsulate paramagnetic MRI contrast agent Gd-DTPA. T1 relaxivity of various liposome formulations was lower than pure Gd-DTPA but with no statistically significant difference (*P* > 0.05). One possible reason could be that the lipid bilayer effectively encapsulated the gadolinium ions and prevented their exchange with water [28]. Besides, the peptide modification on the liposome surface did not alter the integrity of liposome [29]. Another reason might be the hardness of liposome attributable to its components of cholesterol and saturated phospholipids which have low permeability coefficients for water [30]. In this sense, the liposome components merely had a slight influence on the imaging ability of contrast agents Gd-DTPA.

Angiogenesis, the formation of neovessels from existing blood vessel, is a key event in many pathological progresses, especially in the growth invasion and metastasis of tumor [15, 16]. A large number of molecules are involved in the progress of tumor angiogenesis, for instance, VEGF and other factors for vascularization of solid tumors, which involve interaction with membrane *receptors* [17, 31]. One such receptor is neuropilin-1 (NRP1), a co-receptor for VEGFR-2, enhancing the binding and biological activity of VEGF165, which has a wide tissue distribution that includes some tumor-derived cells and endothelial cells [32]. Ala-Thr-Trp-Leu-Pro-Pro-Arg (ATWLPPR), a heptapeptide, has been proved to specifically bind to NRP1 and successfully used for detecting NRP-1positive tumors [12, 17]. However, the relatively low affinity of monomeric A7R indicates further improvement to give a successful imaging [33]. Integrins, one of cell adhesion receptors, also plays a critical role in tumor angiogenesis and metastasis, especially integrin αvβ3, which is highly expressed on tumor cells and activated vascular endothelial cells [34]. The Arg-Gly-Asp amino acid sequence (RGD), which binds specifically to integrinαvβ3 have been broadly used for noninvasive imaging of tumors [7, 21, 27, 35].

During the last decade, simultaneous targeting of multiple receptors is more and more studied in the field of imaging [23, 25, 26, 36]. The TF LP or RGD LP delivery systems, αvβ3, and galectin-1 with paramagnetic Anx/RGD-liposomes have been used for tumor imaging [37, 38]. The synergistic effect of dual-targeted motifs might act through multiple ways. Firstly, the availability of binding sites was a key element of conjugation with peptide ligands. Targeting two receptors simultaneously could increase binding sites on the same cells. Secondly, dual-targeting peptides could bind two different receptors to increase the probability of delivery agents to the interested region. Moreover, link to two different receptor families raising the possibility of binding to heterogeneous tumor cells.

In our previous study, novel dual-targeted paclitaxel entrapped liposomes were successfully constructed by linking a RGD-containing sequence and an ATWLPPR motif with a conjugate with a lys-gly-gly (KGG) spacer and palmitic acid (Pal) anchor and then conjugated to the surface of the liposomes [39]. It revealed that compared to two single-targeted peptides, the dual-targeted peptide had the higher binding activity. These dual-targeted liposomes also maintained a better binding property than the single-targeted formulations.

In the present study, instead of therapy drugs, we encapsulated MRI contrast agent Gd-DTPA in liposomes for molecular imaging. The cellular uptake of targeting paramagnetic liposomes was elevated, and the dual-targeted liposomes indicated higher binding affinity than single-targeted, and, moreover, the mixed single-targeted liposomes. Currently, there are two strategies commonly used for dual-targeting, one is a mixture of two single ligands [25, 38] and the other is combining of two ligands in one molecule [39, 40]. Compared with the utilization of a mixture of individual peptides, we hypothesized that the application of one conjugation coupled with two targets could graft a larger number of peptides per liposome surface. In the competitive binding test, it provided a piece of critical evidence that the effective targeting of the liposome to tumor cells was mediated by the specific binding of ligands and receptors of ανβ3-integrin and neuropilin-1. These data confirmed once again that the RGD-ATWLPPR-combined dual-targeted liposomes facilitated the drug delivery and accumulation in tumor.

In the MR imaging experiment, pure Gd-DTPA and non-targeted liposomes were metabolized rapidly because of their small molecule, water-solubility, and enhanced permeability and retention effects (EPR effects) [41, 42]. In contrast, a prolonged circulating period and accumulation gradually in tumor tissue of dual-targeted liposomes had demonstrated the ability of binding specifically to receptors on tumor cells. P*articularly*, dual-targeted liposomes were more effective than single-targeted liposomes. Dual-targeted liposomes were likely to exert a synergistic effect and the specificity of delivering Gd-DTPA to the tumor site.

In recent years, a great number of dual-targeted nanoparticles have been successfully designed and synthesized for tumor imaging due to their improved binding affinity and specificity. For instance, Wu et al. also used RGD and ATWLPPR motifs to design a dual αvβ3 and NRP-1 targeted heterodimeric peptide for the detection of malignant glioma by positron emission tomography (PET) imaging [43]. In their study, the c (RGDyK) peptide was connected with ATWLPPR through a glutamate linker and then labeled with fluorine-18 (F-18) for radionuclide imaging. In vitro receptor-binding assay demonstrated improved cell uptake and binding affinity of the dual-targeted probe. In addition, in vivo tumor uptake of F-18-labeled dual-RGD-ATWLPPR was significantly higher than that of the single-targeted molecule, and this heterodimeric peptide also had the highest tumor-to-organ ratios. Compared to their radiolabeled peptide probe, our non-radioactive dual-targeted paramagnetic liposomes could deliver contrast agents more effectively to the tumor site due to a greater load capacity. In another study, Zhang et al. successfully constructed 68Ga-BBN (Bombesin)-RGD, a heterodimeric PET tracer targeting both GRPR (gastrin-releasing peptide receptor) and integrin αvβ3, and the clinical data indicated the safety and efficiency of dual-targeting PET radiotracer in prostate cancer diagnosis and staging [44]. However, this dual-targeted PET radiotracer could only be used for noninvasive imaging of prostate cancer because GRPR was an important biomarker for prostate cancer. Unlike the BBN-RGD peptide probe, RGD-ATWLPPR peptide could bind to most of the tumors with the over-expression of VEGFR and/or integrin in the neovasculature of solid tumors. Therefore, this dual-ανβ3-integrin-NRP1-targeting paramagnetic liposome is expected to be used for early detection of various tumors.

## Conclusions

In our study, dual-targeted paramagnetic liposomes were prepared by conjugating two ligands for ανβ3-integrin and neuropilin-1 receptors on the surface and loading MRI contrast agent Gd-DTPA in the core of liposomes. This modification did not significantly interfere with the property of Gd-DTPA. The dual-targeted liposome facilitated the specific cellular uptake in vitro indicating that the affinity and binding of dual-targeted ligand appeared to be synergistically increased. Additionally, in vivo imaging showed that dual peptides-modified liposomes could remain in circulation for a greater portion and longer period than non-targeted or single-targeted counterpart and then exhibit superior selectivity and specificity. To sum up, we successfully constructed a novel angiogenesis-targeting paramagnetic liposome with a dual-targeted heterodimeric peptide which could efficiently bind to the tumor tissue, and we expect these dual-targeted paramagnetic liposomes have the potential to improve the effect of MRI contrast agent for tumor-specific imaging at an early stage.

## Abbreviations

ATWLPPR: Ala-Thr-Trp-Leu-Pro-Pro-Arg
BBN: Bombesin
C6: 6-Aminohexanoic acid
CT: Computed tomography
DMEM: Dulbecco’s Modified Eagle Media
DMSO: Dimethyl sulphoxide
FMOC: Fluorenylmethoxy carbonyl
FOV: Field of view
Gd-DTPA: Gadolinium-diethylenetriamine pentaacetic acid
HPLC: High-performance liquid chromatography
HUVEC: Human umbilical vein endothelial cell
ICP-OES: Inductively coupled plasma optical emission spectrometer
mPEG2000-DSPE: N-(carbonyl-methoxypolyethylene glycol-2000)-1,2-distearoyl-sn-glycero-3-phosphoethanolamine
MRI: Magnetic resonance imaging
NRP1: Neuropilin-1
Pal: Palmitic acid
PBS: Phosphate buffered saline
PC: Phosphatidylcholine
PET: Positron emission tomography
RGD: Arg-Gly-Asp
ROIs: Regions of interest
S/N: Signal-to-noise
SER: Signal enhancement ratio
SI: Signal intensity
STIR: Inversion recovery spin-echo
TE: Echo time
TEM: Transmission electron microscope
TR: Repetition time
VEGF-R: Vascular endothelial growth factor receptor
